# Neuro-glial mechanisms underlying sex-based resilience to cardiorespiratory dysfunction in heart failure

**DOI:** 10.1042/CS20258455

**Published:** 2026-01-14

**Authors:** Katherin V. Pereyra, Karla G. Schwarz, Esteban Díaz-Jara, Sinay C. Vicencio, Liena Bravo, Fernando C. Ortiz, Camilo Toledo, Rodrigo Del Rio

**Affiliations:** 1 Laboratory of Cardiorespiratory Control, Department of Physiology, P. Universidad Católica de Chile, Santiago, Chile; 2 Center for Aging Research and Healthy Longevity, Faculty of Sciences, Universidad Mayor, Santiago, Chile; 3 Mechanisms of Myelin Formation and Repair Laboratory, Departamento de Biología, Facultad de Química y Biología, Universidad de Santiago de Chile, Santiago, Chile; 4 Department of Cell Biology and Physiology, School of Medicine, University of Kansas Medical Center, Kansas City, U.S.A.

**Keywords:** Sex differences, heart failure, RVLM, neuro-glia communication, autonomic imbalance, disordered breathing

## Abstract

Cardiorespiratory disorders, such as sympathoexcitation and disordered breathing, are main hallmarks of chronic heart failure (CHF) associated with high morbidity and mortality. We recently reported that rostral ventrolateral medulla (RVLM) catecholaminergic (C1) neurons are hyperactive in rats with CHF, contributing to cardiorespiratory distress. Notably, CHF without reductions in ejection fraction is more frequent in women than in men, but detailed sex differences in cardiorespiratory pathophysiology, as well as the existence of a sex–dependent neurophysiological remodeling within the RVLM, remain unclear. Therefore, we aimed to characterize sex differences in cardiac, autonomic/respiratory function, RVLM C1 neuron, and glial cell activation, followed by experimentally induced volume overload CHF (with preserved ejection fraction) in adult Sprague–Dawley rats. At week 8 post–CHF induction, male CHF rats showed ˜2.5–fold increased left ventricular dilation, while female CHF rats exhibited only ˜1–fold increase. Unlike the females, both cardiac systolic (end–systolic pressure–volume relationship) and diastolic function (end–diastolic pressure–volume relationship) were depressed in male CHF rats. Indeed, cardiomyocyte calcium handling was impaired only in CHF male rats. Cardiac arrhythmogenesis, sympathoexcitation, and disordered breathing were all significant in CHF male rats but were nearly absent in female rats. Consequently, we found that RVLM C1 neurons were not chronically active in female CHF rats compared with male CHF rats. In addition, we found that male CHF rats showed clear signs of astrocyte reactivity within the RVLM region that results in shifts towards higher astrocyte complexity. The latter was not present in female medullary astrocytes despite showing ventricular hypertrophy. Our results showed that gonadally intact female rats are somehow protected compared with intact male rats from RVLM astrocytic remodeling and chronic C1 neuronal activation following volume overload CHF, being the outcome a less severe cardiorespiratory pathology associated with the CHF phenotype.

## Introduction

Chronic heart failure (CHF) is a multifactorial, life-threatening syndrome with high morbidity and mortality, affecting over 56 million people worldwide [1]. Among its phenotypes, the prevalence of CHF with reduced ejection fraction is expected to remain stable or decline due to improved therapies. In contrast, CHF with preserved ejection fraction (non-ischemic heart disease) is rising steadily and projected to become the most common CHF phenotype [2]. Importantly, marked sex differences exist between these two CHF forms, with the latter (preserved ejection fraction CHF) more prevalent in women [3–5]. This disparity may reflect sex-specific risk factors and pathophysiological responses [4,5]. Women are more prone to concentric left ventricular (LV) remodeling in response to increased afterload and aging [6] and have a more severe diastolic dysfunction than age-matched men [5,6]. While the precise mechanisms underlying these differences are not completely understood, there is evidence that supports a role for reproductive senescence on reduced cardio-protection contributing to increased risk for developing CHF in women [7]. Interestingly, adverse cardiovascular events are rare before menopause [8]. Despite the physiological/cellular/molecular mechanisms that offer cardiovascular protection at younger ages, before reproductive senescence, these mechanisms remain unresolved.

Altered cardiovascular and respiratory reflexes, such as reduced baroreflex sensitivity and enhanced chemoreflex sensitivity, drive sympatho-vagal imbalance and disordered breathing in CHF [7,8]. Chronic sympathoexcitation, often originating from central autonomic dysfunction, promotes adverse cardiac remodeling, increased LV stiffness, and arrhythmogenesis [9,10]. Whether these reflex alterations differ by sex remains unclear. Furthermore, whether differences in the neural control of cardiorespiratory regulation (between males and females) confer protection or further compromise cardiac function deterioration in the setting of CHF is not known.

Pre-sympathetic catecholaminergic (C1) neurons in the rostral ventrolateral medulla (RVLM) provide major excitatory input to spinal sympathetic preganglionic neurons. Increased RVLM activity is implicated in CHF progression [11,12], but the mechanisms underlying C1 hyperactivity remain unclear. Activated astrocytes in the RVLM can stimulate bulbospinal sympathoexcitatory neurons, elevating renal sympathetic nerve activity and blood pressure (BP) [13]. We previously demonstrated that male rats with CHF display hallmark features of sympathoexcitation and sleep-disordered breathing, with RVLM C1 neurons driving elevated sympathetic tone, respiratory instability, cardiac decompensation, and sudden death [11,14–16]. Despite well-documented sex differences in CHF, women remain underrepresented in cardiovascular clinical trials [17,18], and preclinical research has rarely addressed sex-specific mechanisms in the control of cardiorespiratory function. Moreover, whether changes in the neural control of autonomic and respiratory function differ between males and females in the setting of CHF is completely unknown. In the present study, we hypothesized that adult, gonadally intact female rats exhibit a more favorable cardiac and cardiorespiratory response to volume overload-induced CHF than males, reflecting sex-specific protective mechanisms that limit pathological remodeling within central autonomic circuits. Accordingly, we investigated sex differences in cardiac autonomic function, disordered breathing, chemoreflex sensitivity, and LV systolic and diastolic function in an experimental rat model of CHF, without the confounding factor of reproductive senescence.

## Methods

### Ethical considerations and animals

Adult female and male Sprague–Dawley rats (*n* = 50) weighing 230 ± 12 g were housed in the animal vivarium from the University of Kansas Medical Center and the Pontificia Universidad Católica de Chile in 12-h light–dark control rooms with *ad libitum* access to food and water. Experimental protocols were approved by the IACUC from the University of Kansas Medical Center (24-02-378) and the P. Universidad Católica de Chile (170914006) and were conducted according to National Institutes of Health Guidelines for the Care and Use of Laboratory Animals. Animals were randomly assigned to the following experimental groups: (i) Sham male, (ii) CHF male, (iii) Sham female, and (iv) CHF female (Supplementary Figure S1A). At the end of the experimental protocol, all animals were humanely euthanized with an overdose of anesthesia (sodium pentobarbital 100 mg/Kg i.p.).

### CHF model

CHF was induced by surgical creation of an arteriovenous fistula as previously described [11,15,16,19]. Under sterile conditions, a laparotomy was performed under isoflurane anesthesia (5% induction, 1.5% maintenance, balanced with O₂) to isolate the inferior vena cava and abdominal aorta. Both vessels were clamped caudal to the renal artery and rostral to the femoral arteries. The aorta was punctured with an 19-gauge needle and advanced until perforating the adjacent vena cava. A drop of Histoacryl glue (B. Braun, Germany) sealed the puncture site. Fistula patency was visually confirmed by arterial pulsatile flow into the venous circulation. The peritoneal cavity and skin were closed with absorbable sutures (Novosyn 4/0 and Novosyn 3/0, B. Braun, Germany). Postoperative care included antibiotic (enrofloxacin 10 mg/kg i.p., seven days), meloxicam (2 mg/kg i.p., three days), and saline (3 mL 0.9% NaCl as needed). Sham rats underwent the same procedure without fistula creation.

### Echocardiography measurement

Four and eight weeks after CHF induction, LV dimensions were assessed via transthoracic echocardiography under isoflurane anesthesia (5% induction; 1.5% maintenance balanced with 100% O₂) (Supplementary Figure S1). CHF rats were confirmed by a ≥1.5-fold increase in LV end-diastolic volume (LVEDV) and stroke volume (SV) compared with Sham, without changes in ejection fraction (EF) at week 4 post-CHF induction [11,15,16]. M-mode echocardiography was performed at the mid-papillary muscle level in the parasternal short-axis view (Mindray Z6 Vet). LV end-systolic diameter (LVESD) and LVEDV were averaged from three consecutive cardiac cycles [11,16]. LV end-systolic volume (LVESV) and LVEDV were calculated using the Teichholz method: [ESV = 7 × ESD³/(2.4 + ESD)] and [EDV = 7 × EDD³/(2.4 + EDD)], respectively. EF and fractional shortening were derived from LV volumes and diameters [20].

### Radiotelemetry implant surgery

Arterial BP and heart rate (HR) were recorded in conscious rats using radiotelemetry (Data Science International, U.S.A.). Devices were implanted one week before physiological testing (week 7 post-CHF induction). Under isoflurane anesthesia (1.5–2% in O₂), a skin incision in the right leg exposed the femoral artery. The pressure transducer tip (HD-S10) was inserted into the artery, and the transmitter body was placed subcutaneously [11,21]. Postoperative care included enrofloxacin (10 mg/kg i.p., seven days) and meloxicam (2 mg/kg i.p., three days). BP was sampled at 500 Hz and recorded between 9 am and 12 pm, once animals were resting in eupneic normoxic conditions. HR was derived from the 1st derivative of blood pressure ( dP/dt) signal, and mean arterial blood pressure, systolic blood pressure, diastolic blood pressure, and pulse pressure were calculated from BP traces.

### Cardiac autonomic function and arrhythmia incidence

Autonomic balance was assessed by pharmacological blockade in conscious rats. Propranolol (1 mg/kg i.p.), a β-adrenergic antagonist, was used to estimate sympathetic drive/tone to the heart. HR changes (ΔHR) were calculated relative to baseline. Tachograms were generated from dP/dt data over 30 min, and arrhythmias were quantified (premature or delayed beats with intervals >2.5 standard deviations from the mean). Movement artifacts were removed. The total number of arrhythmic events per 30 min was reported.

### Resting breathing and ventilatory chemoreflex

Respiratory parameters during normoxia and chemoreflex activation were measured in unrestrained rats by whole-body plethysmography (EMKA Technologies, France) and analyzed with ecgAUTO software [16,22]. Rats were habituated to the chamber for three days prior to the acquisition of respiratory signals. Resting breathing was recorded for 2 h in room air; only the final hour was analyzed to ensure sleep (confirmed by telemetry). Breathing regularity was assessed via Poincaré plots from ~400-breath intervals. The coefficient of variation of tidal volume (V_T_) was calculated. The irregularity score (IS) was computed as 100 × (TTOT_n_ − TTOT_n_₋₁)/TTOT_n_₋₁, where TTOT_n_ is the duration of the n^th^ breath.

Since we previously found evidence of heightened central chemoreflex drive following volume overload, we tested central chemoreflex sensitivity by allowing the rats to breathe a hypercapnic gas mixture (F_i_CO_2_ 7%) while resting inside the plethysmograph chambers. Responses were expressed as the slope of the change from baseline to hyperventilation, yielding hypercapnic ventilatory responses (HCVRs).

### Intraventricular pressure–volume loops

LV hemodynamics were assessed at week 8 post-CHF induction. Rats were anesthetized with α-chloralose (40 mg/kg i.p.) and urethane (800 mg/kg i.p.), intubated, and instrumented with a PV conductance catheter (Millar SPR-869, U.S.A.) via the right carotid artery [23,24]. The inferior vena cava was briefly occluded to assess contractility [end-systolic pressure–volume relationship (ESPVR)] and relaxation [end-diastolic pressure–volume relationship (EDPVR)] independent of volume. Baseline parameters were averaged from 20 consecutive loops. Volumes were calibrated by the cuvette method and NaCl 30% i.v. bolus for parallel conductance. Data were acquired at 1 kHz (Power Lab Model 1645, ADInstruments) and analyzed in LabChart v7.3.8 (ADInstruments).

### *Ex vivo* adult cardiomyocyte imaging

Cardiomyocytes were isolated from 8 weeks CHF rats for functional studies. Cardiomyocyte isolation buffer (CIB; composition in mM: 120 NaCl, 5.4 KCl, 0.5 MgSO₄•7H₂O, 0.33 Na₂HPO₄•2H₂O, 25 HEPES, 30 taurine, 22 glucose anhydro, 10 butanedione monoxime) was prepared 24 h prior and stored at 4°C. Rats were heparinized (1000 U/kg i.p.) 20 min before thoracotomy under α-chloralose/urethane anesthesia. Hearts were excised, immersed in ice-cold perfusion buffer (PB; CIB + 0.4 mM EGTA), cannulated via the aorta, and perfused on a Langendorff system at 37°C, 5 ml/min for 1 min. Hearts were then digested for 15 min at 37°C in digestion buffer (DB; CIB + 300 µM CaCl₂, 0.2 mg/mL protease XIV, 2.4 mg/mL collagenase II). The LV was dissected, minced, and incubated in DB for 5 min and then transferred to stop buffer (SB; CIB + 5% FBS, 1.5 mM CaCl₂). Cells were allowed to settle, washed, and plated on laminin-coated coverslips at 37°C, 5% CO_2_. Calcium transients were recorded in Fluo-4–loaded cells (5 µM) in recording buffer (RB; in mM: 120 NaCl, 5.4 KCl, 1 MgCl₂, 25 HEPES, 1.1 CaCl₂) using a Zeiss 710 confocal microscope in line-scan mode during 1 Hz electrical stimulation. Images were analyzed in ImageJ.

### Western blot

RVLM micropunches were lysed in RIPA buffer (Sigma–Aldrich) with 1% protease inhibitor cocktail. Protein content was measured by BCA assay. Equal protein amounts (30 µg) were separated by SDS–PAGE (10% gel), transferred to PVDF membranes, and blocked in TBS-T (0.1%) + 5% milk. Membranes were incubated overnight at 4°C with anti-FosB (rabbit, 1:1000, Cell Signaling 2251), washed, and incubated with HRP-conjugated anti-rabbit secondary (1:2000, KPL). Bands were visualized with SuperSignal West Femto (Thermo Fisher). Membranes were stripped, re-blocked, and reprobed with anti-GAPDH (mouse, 1:2000, Santa Cruz sc-32233) and HRP-conjugated anti-mouse secondary (1:2000, KPL) (Supplementary Figure S2).

### Immunofluorescence and Sholl analysis

Rats were anesthetized with sodium pentobarbital (100 mg/kg i.p.) and perfused with 0.9% NaCl followed by 4% paraformaldehyde. Brains were post-fixed overnight, cryoprotected in 30% sucrose, and sectioned coronally at 25 µm. Free-floating sections were blocked (3% BSA, 0.5% Triton X-100 in PBS) for 1 h, incubated overnight with anti-tyrosine hydroxylase (rabbit, 1:1000) and anti-GFAP (mouse, 1:1000), washed, and incubated with Alexa Fluor 488- and 555-conjugated secondary antibodies (Invitrogen) for 2 h. Nuclei were stained with Hoechst 33,342 (1:1000, 10 min). Sections were mounted in fluorescent mounting medium and imaged on a Zeiss LSM 800 confocal microscope (40 × oil, 1.4 NA) with 0.5 µm z-steps. Images were processed in ImageJ.

Sholl analysis was used to assess astrocyte morphological features. Only cells with intact processes within the RVLM region were used for analysis [25]. Concentric spheres at 1 µm intervals from the soma were used to count process intersections. Six morphometric parameters (branch length, path length, branch points, paths, total cable length, convex hull area) were extracted from 3D reconstructions (Simple Neurite Tracer, Fiji). Analyses were performed blinded to group.

### Multivariable correlations, principal component analysis, and K-means clustering

Morphological data (n astrocytes × 6 variables) were standardized using z-scores: zᵢ = (xᵢ − µ)/σ [26]. Pearson correlations were visualized in a clustered heatmap. Principal component analysis (PCA) was performed on the correlation matrix; components with eigenvalues > 1 were retained [27]. Scores for PC1 and PC2 were plotted, and variable loadings were calculated. K-means clustering (k = 5) was applied to PC1–PC2 scores, based on reported RVLM astrocyte heterogeneity [28]. Clusters were regrouped into low, medium, or high morphological complexity. The proportion of astrocytes in each group was calculated for each condition. Analyses were performed in Python (v3.10) using pandas, scikit-learn, matplotlib, and seaborn.

### Statistical analysis

Data are presented as mean ± SEM (text, tables) and min–max box plots (figures). One-way ANOVA followed by Holm–Sidak *post hoc* test was used for group comparisons. Analyses were performed with GraphPad Prism 8.0 (GraphPad Software Inc., La Jolla, CA, U.S.A.).

## Results

### Arteriovenous fistula induces enlarged LV in both male and female groups

**Figure 1:**
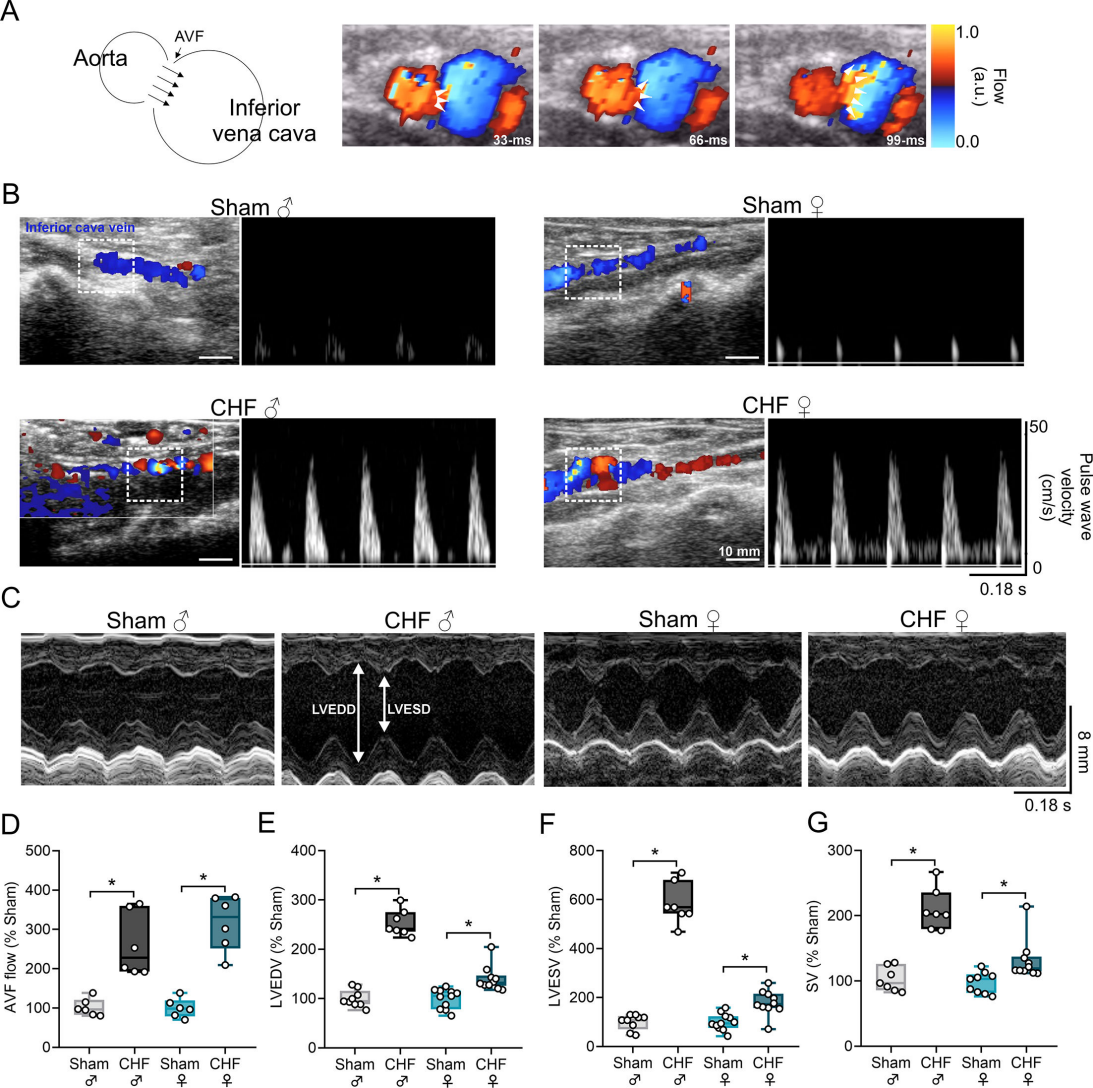
Female rats present lower left ventricular hypertrophy induced by arteriovenous fistula. (**a**) Schematic representation of arteriovenous shunt to induce volume overload and a representative color Doppler ultrasound of an arteriovenous fistula (AVF). Note the arterial blood flow infiltrating into the cava vein. (**b**) Representative pseudo-color images of Doppler and pulse-wave Doppler showing blood flow velocities across the inferior vena cava and abdominal aorta of each experimental group. Note that CHF rats that underwent arteriovenous shunt showed arterial blood flowing into the venous circulation. (**c**) Representative M-mode echocardiography of left ventricle (LV) from one male Sham, one male CHF, one female Sham, and one female CHF rats. White arrows show LV end-diastolic diameter (LVEDD) and LV end-systolic diameter (LVESD). (**d-g**) Summary data of echocardiographic parameters: AVF flow, LV end-diastolic volume (LVEDV), LV end-systolic volume (LVESV), stroke volume (SV). Data were analyzed using One-way ANOVA and Holm–Sidak multiple comparisons test; *: *P*<0.05. *n* = 7–11 per group.

A high-output CHF model was generated by creating an arteriovenous shunt between the abdominal aorta and vena cava, producing a chronic preload increase (Figure 1a). Pulse-wave Doppler confirmed pulsatile arterial flow into the venous return (Figure 1a and b). Female CHF surgeries followed the same protocol as in males, yielding comparable pulsatile flow in the vena cava (Figure 1b and d) and LV chamber enlargement (Figure 1c; Table 1). Transthoracic M-mode echocardiography showed significantly increased LV end-diastolic diameter (LVEDD) and LVESD in both CHF groups versus respective Shams (Table 1). The magnitude differed by sex: male CHF rats exhibited ~2.5- and ~ 5-fold increases in LVEDV and LVESV, respectively (vs. male Sham), whereas female CHF showed ~1-fold increases (Figure 1e and f). SV increased in both CHF groups versus Sham but was lower in females than males (Figure 1g).

**Table 1:** Global hemodynamic parameters

	Sham ♂ (*n* = 8)	CHF ♂ (*n* = 7)	Sham ♀ (*n* = 11)	CHF ♀ (*n* = 10)
LVEDD (mm)	5.9 ± 0.1	8.9 ± 0.16*	5.8 ± 0.2	6.8 ± 0.2†
LVESD (mm)	2.4 ± 0.1	5.1 ± 0.1	2.7 ± 0,2	3.6 ± 0.2
LVEDV (µl/100 g)	17.6 ± 0.9	44.7 ± 1.8*	17.3 ± 1.0	24.3 ± 1.4†
LVESV (µl/100 g)	2.1 ± 0.2	12.1 ± 0.7*	3.2 ± 0.3	5.6 ± 0.5
SV (µl/100 g)	15.6 ± 0.9	32.6 ± 1.9*	14.2 ± 0.7	18.7 ± 1.4
EF (%)	88.2 ± 1.2	72.7 ± 1.7*	83.4 ± 1.9	76.7 ± 2.1
FS (%)	59.4 ± 1.5	42.9 ± 1.4*	53.7 ± 2.2	47.0 ± 2.2
BW (g)	500.4 ± 4.7	491.6 ± 16.7*	282.4 ± 6.0	303.2 ± 8.3†

Values are means ± S.E.M. Two-way ANOVA followed by Holm–Sidak *post hoc* analysis. *P<0.05 vs. Sham male, †*P*<0.05 vs. Sham female.

LVEDD, left ventricular diastolic diameter. LVESD, left ventricular systolic diameter. LVEDV, left ventricular end-diastolic volume. LVESV, left ventricular end-systolic volume. SV, stroke volume. EF, ejection fraction. FS, fractional shortening. BW , body weight.

### Female CHF rats display normal left ventricular systolic and diastolic function compared with male CHF rats

**Figure 2:**
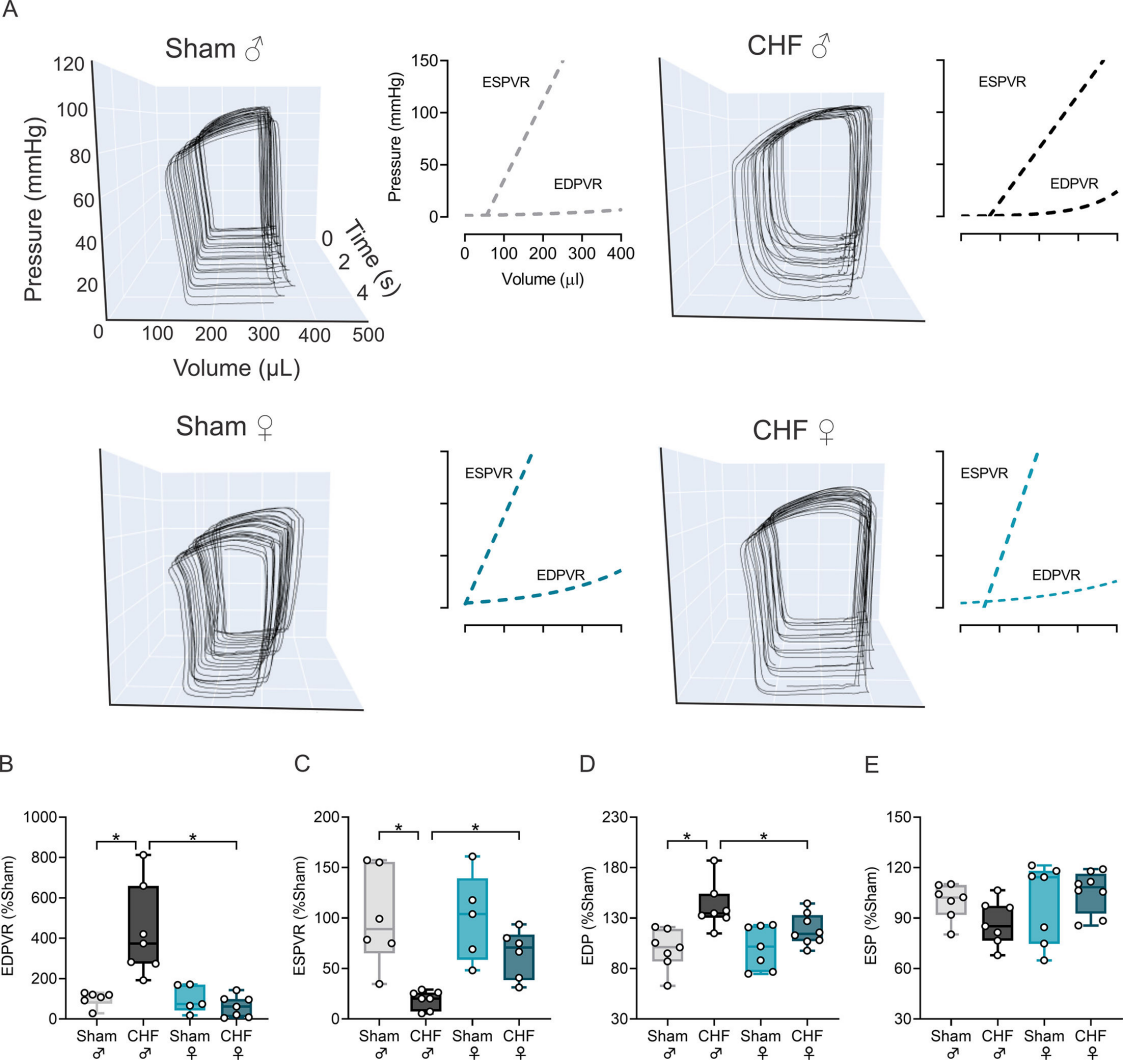
Female rats subjected to volume-overload CHF maintained LV systolic and diastolic function. (**a**) Right panel: Representative 20 consecutive intraventricular pressure–volume (PV) loops during vena cava occlusion to assess load-independent indexes of cardiac function. Left panel: Representative end-systolic and end-diastolic PV relationships (ESPVR and EDPVR), characterizing LV systolic and diastolic properties, respectively. Note that ESPVR is a regression between multiple correlated end-systolic volume (ESV) and end-systolic pressure (ESP) points, while EDPVR is non-linear and reflects LV compliance. (**b-e**) Summary data showing end-diastolic pressure (EDP) and end-systolic pressure (ESP), ESPVR, and EDPVR, respectively. Data were analyzed using One-way ANOVA and Holm–Sidak multiple comparisons test; *: *P*<0.05. *n* = 7–8 per group.

To further explore differences in cardiac (dys)function between male and female CHF, we performed ventricular pressure–volume loops using intraventricular catheterization (Figure 2). Baseline intraventricular parameters are presented in Table 2. Contractile function was impaired in male CHF, evidenced by decreases in ESPVR (0.41 ± 0.08 vs. 0.08 ± 0.01 mmHg/µL, Sham male vs. CHF male) (Figure 2a–c). Interestingly, ESPVR remained unchanged in the CHF female compared with the Sham female (1.08 ± 0.21 vs. 0.70 ± 0.11 mmHg/µL, Sham female vs. CHF female). Similarly, LV compliance was also affected in the CHF male but not in the CHF female group, as evidenced by changes in EDPVR. Indeed, male CHF showed significant diastolic impairment (EDPVR: 0.005 ± 0.0001 vs. 0.02 ± 0.004 mmHg/µL, Sham male vs. CHF male), while female CHF remained like Sham female (EDPVR: 0.007 ± 0.002 vs. 0.005 ± 0.001 mmHg/µL, Sham female vs. CHF female) (Figure 2a and c). Accordingly, male CHF rats display increased end-diastolic pressure (EDP) compared with male Sham (3.88 ± 0.33 vs. 5.69 ± 0.29 mmHg, Sham male vs. CHF male) (Figure 2d), suggesting impaired ventricular relaxation. EDP was unaltered in CHF females (3.61 ± 0.37 vs. 4.27 mmHg, Sham female vs. CHF female) (Figure 2d). Non-significant changes were found in end-systolic pressures (ESP) between all groups (Figure 2e).

**Table 2:** PV loops cardiac function parameters

	Sham ♂ (*n* = 8)	CHF ♂ (*n* = 7)	Sham ♀ (*n* = 11)	CHF ♀ (*n* = 10)
***Global cardiac function***				
HR (bpm)	389.9 ± 25.8	361.3 ± 25.1^ǂ^	316.9 ± 18.2	276.4 ± 13.9
CO (mL/min/100 g)	12.9 ± 1.6	23.6 ± 3.2*	12.1 ± 1.8	17.8 ± 1.6
***Preload and afterload***				
LVEDV (µL/100 g)	50.2 ± 3.5	89.7 ± 6.5*	71.5 ± 10.4	90.8 ± 6.6
Ea (mmHg/ µL)	0.6 ± 0.1	0.3 ± 0.01^ǂ^*	1.0 ± 0.1	0.6 ± 0.1^α^
***Systolic function***				
LVEF (%)	73.6 ± 6.3	77.8 ± 4.9	72.3 ± 4.9	76.9 ± 4.4
LVESV (µL/100 g)	16.9 ± 2.7	25.5 ± 2.6*	33.9 ± 9.3	26.9 ± 4.3
dP/dt max (mmHg/s)	10349 ± 1981	9211 ± 1058	8718 ± 1058	9879 ± 592
***Diastolic function***				
Tau (ms)	7.5 ± 0.7	8.3 ± 0.6	12.6 ± 0.9	13.4 ± 0.7
dP/dt min (mmHg/s)	-9774 ± 917	-6102 ± 695	-5929 ± 981	-5795 ± 620

Values are expressed as mean ± S.E.M. Tau, time constant of relaxation. ^ǂ^ *P*<0.05 vs. CHF female. *P<0.05 vs. Sham male.^ α^ *P*<0.05 vs. Sham female.

HR , heart rate. CO, cardiac output. LVEDV, left ventricle end-diastolic volume. Ea, elastance. LVEF, left ventricle ejection fraction. LVESV, left ventricle end-systolic volume.

### Calcium handling in ventricular myocytes is impaired only in male rats with CHF

**Figure 3:**
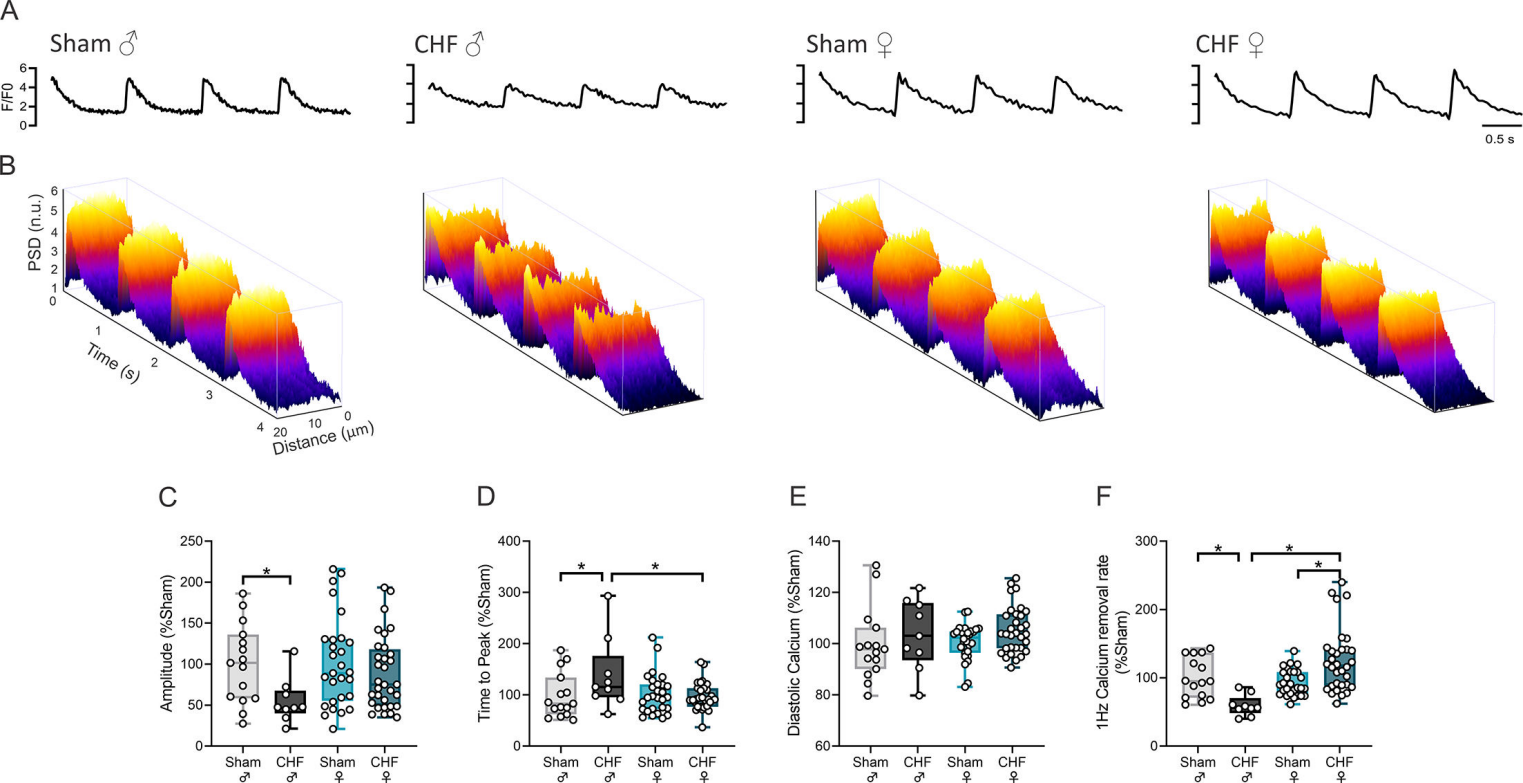
Female CHF rats display normal diastolic kinetic and bioavailability of cytosolic Ca2^2+^ transients in cultured cardiomyocytes. (**a**) Representative line-scan recordings of electrically stimulated cytosolic Ca^2+^ transients in isolated cardiomyocytes of one male Sham, one male CHF, one female Sham, and one female CHF rats, and (**b**) power spectral density (PSD) from those representatives. Note that male CHF rats show impaired systolic Ca^2+^. (**c-f**) Summary data showing calcium removal rate, calcium dye fluorescence amplitude, time to peak, and diastolic calcium, respectively. Data were analyzed using One-way ANOVA and Holm–Sidak multiple comparisons test; *: *P*<0.05. Sham male, *n* = 4 (15 cells); CHF male, *n* = 3 (9 cells); Sham female, *n* = 3 (28 cells); CHF female, *n* = 4 (32 cells).

We aimed to evaluate changes at the single-cell level by assessing calcium handling in isolated cardiomyocytes. Therefore, we assessed electrically evoked calcium transients in cardiomyocytes obtained from the left ventricle (Figure 3; Table 3). We found that male CHF cardiomyocytes display both systolic and diastolic calcium management impairment, which parallels the whole organ physiology when compared with previous results of ESPVR and EDPVR. First, the amplitude of the Ca^2+^ response decreased ~1.8 fold compared with male Sham (CHF male: 54.0 ± 8.3% Sham) (Figure 3a–c), while female CHF cardiomyocytes remained within the range observed in the female Sham group. Notably, the time to calcium peak was increased by ~1.4-fold in CHF male cardiomyocytes (CHF male: 139.0 ± 23.6% Sham) (Figure 3d). No differences in diastolic calcium between experimental groups were found (Figure 3e). Calcium re-uptake rate is significantly reduced in male CHF (59.31 ± 5.09% Sham, CHF male) (Figure 3f).

**Table 3:** In-vitro isolated cardiomyocyte calcium imaging parameters

	Sham ♂ (*n* = 4)	CHF ♂ (*n* = 3)	Sham ♀ (*n* = 4)	CHF ♀ (*n* = 3)
Calcium removal rate 1 Hz (1/tau)	8.1 ± 0.6	4.8 ± 0.4*^ǂ^	5.2 ± 0.3	6.5 ± 0.4^α^
Systolic calcium (F/F0)	3.7 ± 0.3	2.5 ± 0.2*	3.5 ± 0.2	3.3 ± 0.2
Amplitude (∆F/F0)	2.7 ± 0.1	1.5 ± 0.2*	2.6 ± 0.2	2.3 ± 0.2
Diastolic calcium (F/F0)	0.9 ± 0.03	1.0 ± 0.04	1.0 ± 0.01	1.0 ± 0.02
Tau (ms)	0.1 ± 0.01	0.2 ± 0.02*	0.2 ± 0.01	0.2 ± 0.01

Values are expressed as mean ± S.E.M. ^ǂ^ *P*<0.05 vs. CHF female. *P<0.05 vs. Sham male.^ α^ *P*<0.05 vs. Sham female. Sham male 15 cells, CHF male 9 cells, Sham female 28 cells, CHF female 32 cells.

### Cardiac sympathetic drive and arrhythmia susceptibility are enhanced in male CHF but are completely absent in female CHF rats

**Figure 4:**
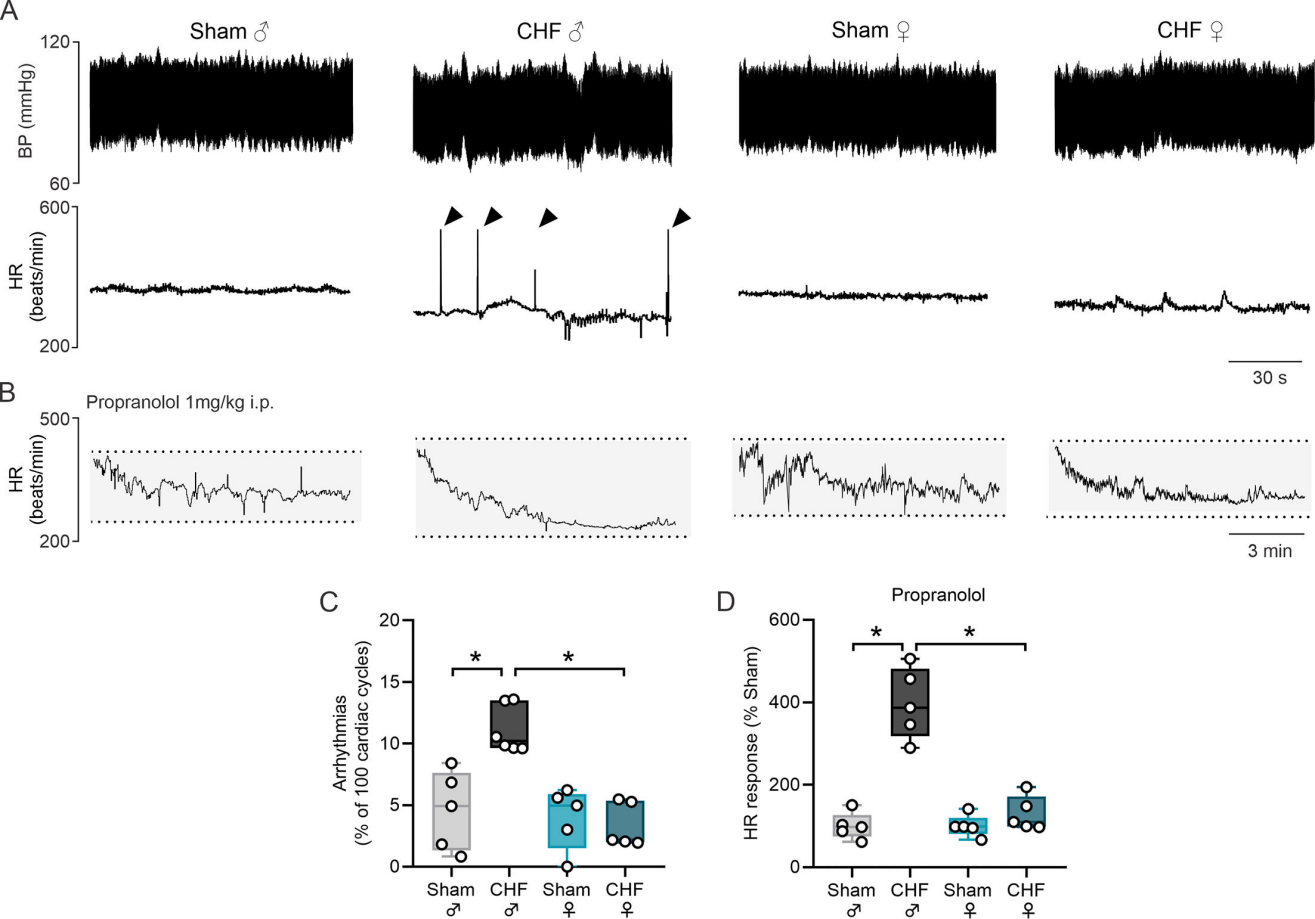
CHF induction in female rats failed to trigger cardiac autonomic imbalance and arrhythmias. (**a**) Representative traces of resting blood pressure–first derivative (dP/dt, upper panel) and heart rate (HR, bottom panel) in one male Sham, one male CHF, one female Sham, and one female CHF rat. Arrowheads show ventricular arrhythmic events. (**b**) Representative HR traces following acute administration of propranolol (1 mg/kg i.p., upper panel) and acute administration of atropine (1 mg/kg i.p., bottom panel) to evaluate cardiac sympathetic tone and parasympathetic tone, respectively. Note that the male CHF rat showed a larger response to propranolol and a decreased response to atropine compared with the female CHF rat. (**c,d**) Summary data showing resting basal HR and arrhythmia incidence, respectively. (**e,f**) Summary data showing the chronotropic response as ΔHR to propranolol and atropine, respectively. Data were analyzed using One-way ANOVA and Holm–Sidak multiple comparisons test; *: *P*<0.05. *n* = 5–6 per group.

Diastolic dysfunction and calcium handling impairment are associated with alterations in cardiac rhythm and cardiac autonomic imbalance. Male CHF rats showed decreased HR at rest (315.7 ± 9.5 vs. 273.3 ± 12.3, Sham male vs. CHF male) (Table 4), an increase in the number of ventricular arrhythmias (4.6 ± 1.3 vs. 12.2 ± 0.9 events/30 min, Sham male vs. CHF male) (Table 4, Figure 4a and c) and a ~4-fold increase in cardiac sympathetic drive (∆HR to propranolol: -24.5 ± 2.0 vs. -97.4 ± 9.4 bpm; Sham male vs. CHF male) (Figure 4b and d). Notably, CHF females showed no signs of cardiac arrhythmogenesis (3.8 ± 1.1 vs. 3.2 ± 0.7 events/30 min, Sham female vs. CHF female) (Figure 4a and c), nor evidence of heightened cardiac sympathetic drive (Figure 4b and d). Indeed, cardiac chronotropic responses to propranolol administration in CHF female rats were indistinguishable from the ones obtained in Sham female rats (∆HR: -16.76 ± 2.01 vs. -23.03 ± 3.67 bpm, Sham female vs. CHF female) (Figure 4b and d). No differences were found in arterial BP between experimental groups (Table 4).

**Table 4:** Baseline blood pressure and heart rate values

	Sham ♂ (*n* = 5)	CHF ♂ (*n* = 5)	Sham ♀ (*n* = 5)	CHF ♀ (*n* = 6)
SBP (mmHg)	121.7 ± 7.2	110.0 ± 4.00	107.6 ± 3.1	106.2 ± 3.8
DBP (mmHg)	89.9 ± 5.3	82.7 ± 3.5	83.1 ± 2.8	80.8 ± 3.2
MABP (mmHg)	103.4 ± 4.2	94.3 ± 3.5	91.3 ± 2.8	89.2 ± 3.4
PP (mmHg)	31.0 ± 3.2	27.3 ± 2.8	24.5 ± 1.6	25.4 ± 1.1
HR (bpm)	315.7 ± 9.5	273.3 ± 12.3*	320.4 ± 6.7	311.4 ± 9.5

Values are mean ± S.E.M. Two-way ANOVA followed by Holm–Sidak *post hoc* test. *P<0.05 vs. Sham male. Sham female.

MABP, mean arterial blood pressure. SBP, systolic blood pressure. DBP, diastolic blood pressure. PP, pulse pressure. HR, heart rate.

### RVLM neuronal and astrocyte activation in the setting of CHF

**Figure 5:**
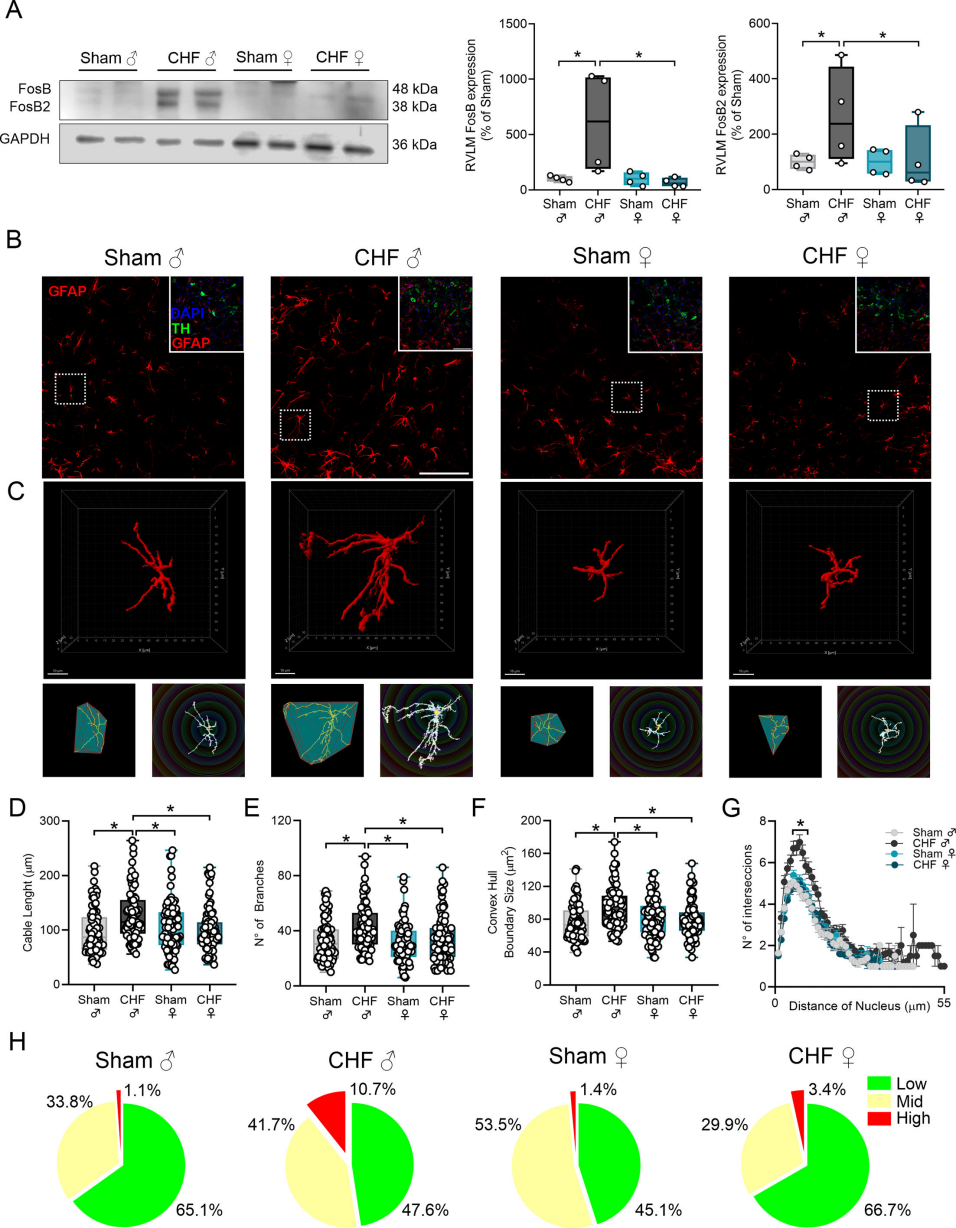
Male CHF rats but not female CHF rats displayed RVLM chronic neuronal activation and remodeling of astrocyte complexity. (**a**) Representatives immunoblot against FosB transcription factor and quantification of FosB and FosB2 protein expression of RVLM micropunches. (**b**) Representative confocal images showing GFAP^+^ astrocytes in male and female Sham and CHF rats. In sets: higher magnification with GFAP (red), TH (green), and DAPI (blue). Scale bar = 100 μm (**c**) 3D reconstructions and skeletal representations of single GFAP^+^ astrocytes per group, with convex hull and Sholl topology visualization. (**d-e**) Summary data showing quantification of astrocyte cable length, number of branches, and convex hull boundary size, respectively. (**g**) Number of intersections as a function of the distance from the nucleus. (**h**) Pie charts showing the proportion of astrocytes in each morphological complexity category (low, medium, high) derived from unsupervised clustering on PCA-reduced morphometric data. Data were analyzed using One-way ANOVA and Holm–Sidak multiple comparisons test; *: *P*<0.05. *n* = 80–100 cells per group; *n* = 3–4 animals per group.

RVLM is a major nodal point of the central pre-sympathetic network and is thought to contribute to increased sympathetic outflow in heart disease; chronic neuronal activation of pre-sympathetic motoneurons from the RVLM has been shown in rats with CHF [12,16]. We found that male CHF animals showed greater RVLM expression of FosB and FosB2 proteins (i.e. neuronal markers of chronic activation) compared with Sham male animals (FosB: 100.0 ± 17.9 vs. 608.6 ± 240.4% Sham; FosB2: 100.0 ± 17.1 vs. 261.1 ± 87.6% Sham, Sham male vs. CHF male) (Figure 5a). On the contrary, no evidence of chronic neuronal activation was found in RVLM micropunches obtained from female CHF rats (Figure 5a). Since enhanced astrocyte (re)activity in the RVLM is strongly associated with impaired autonomic regulation through neuron-glia crosstalk [29], we evaluated RVLM astrocyte morphology complexity as a proxy of astrocyte reactivity [30]. Compared with male Sham animals, CHF male animals exhibited an increase in the astrocyte cell marker GFAP in the RVLM (Figure 5b), whereas no difference was observed between Sham and CHF females (Figure 5b). In addition, 3D reconstruction of medullary astrocytes revealed a higher degree of complexity in male CHF rats compared with male Sham rats. Indeed, CHF male RVLM astrocytes displayed augmented branch length, branch number, and convex hull volume compared with male Sham RVLM astrocytes (cable length: 97.52 ± 4.37 vs. 140.10 ± 7.25 μm; # of branches: 33.60 ± 1.77 vs. 48.92 ± 3.31; convex hull boundary size: 77.08 ± 2.57 vs. 95.83 ± 2.97 μm^2^, Sham male vs. CHF male; Figure 5d–f). Contrarily, GFAP^+^ staining in the RVLM of CHF females was significantly lower compared with CHF males and showed no difference when compared with Sham Female RVLM astrocytes (Figure 5b).

To explore underlying morphological diversity on RVLM astrocytes, we applied unsupervised multivariate analysis. We identified five distinct structural clusters of RVLM astrocytes within the four experimental conditions (Supplementary Figure S3). We regrouped these five clusters *post hoc* into three broader categories of morphological complexity (low, medium, and high) as previously described [31]. We then examined the distribution of these complexity categories across experimental groups (Figure 5h). Sham animals (from both sexes) displayed a high proportion of RVLM astrocytes showing low and medium complexity, with only a smaller fraction in the high complexity category (1.1% and 1.4%, Sham male and Sham female, respectively). Interestingly, we found that CHF results in a pronounced morphological reorganization of RVLM astrocytes, specifically towards increased structural complexity, but only in male CHF rats. Indeed, a ~10-fold increase in the proportion of high complex RVLM astrocytes was found in males but not in female CHF rats (Figure 5h).

### CHF female rats showed no signs of disordered breathing

**Figure 6:**
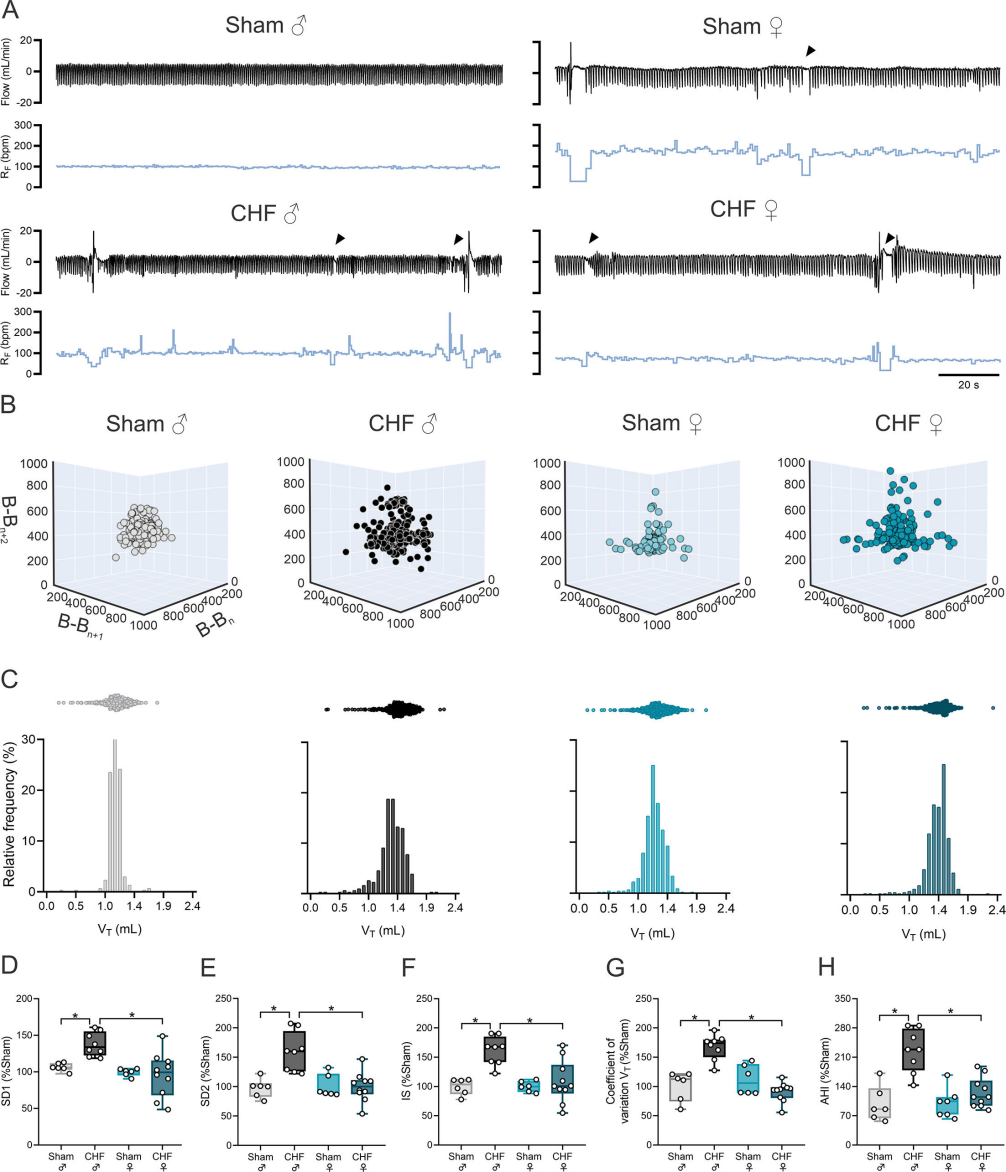
Female CHF rats showed no signs of disordered breathing at rest. (**a**) Representative ventilatory flow recordings (top) and frequency (bottom) obtained from one male Sham, one male CHF, one female Sham, and one female CHF rat. (**b**) Representative 3D Poincaré plots displaying breath-to-breath interval variability of one rat per group. (**c**) Frequency histograms showing the distribution of tidal volume (V_T_) cycle amplitudes. (**d,e**) Summary data showing short-term (SD1) and long-term (SD2) breathing interval variability. (**f,g**) Summary data showing the coefficient of variation of V_T_ and irregularity score (IS). Data were analyzed using One-way ANOVA and Holm–Sidak multiple comparisons test; *: *P*<0.05. *n* = 6–10 per group.

**Figure 7:**
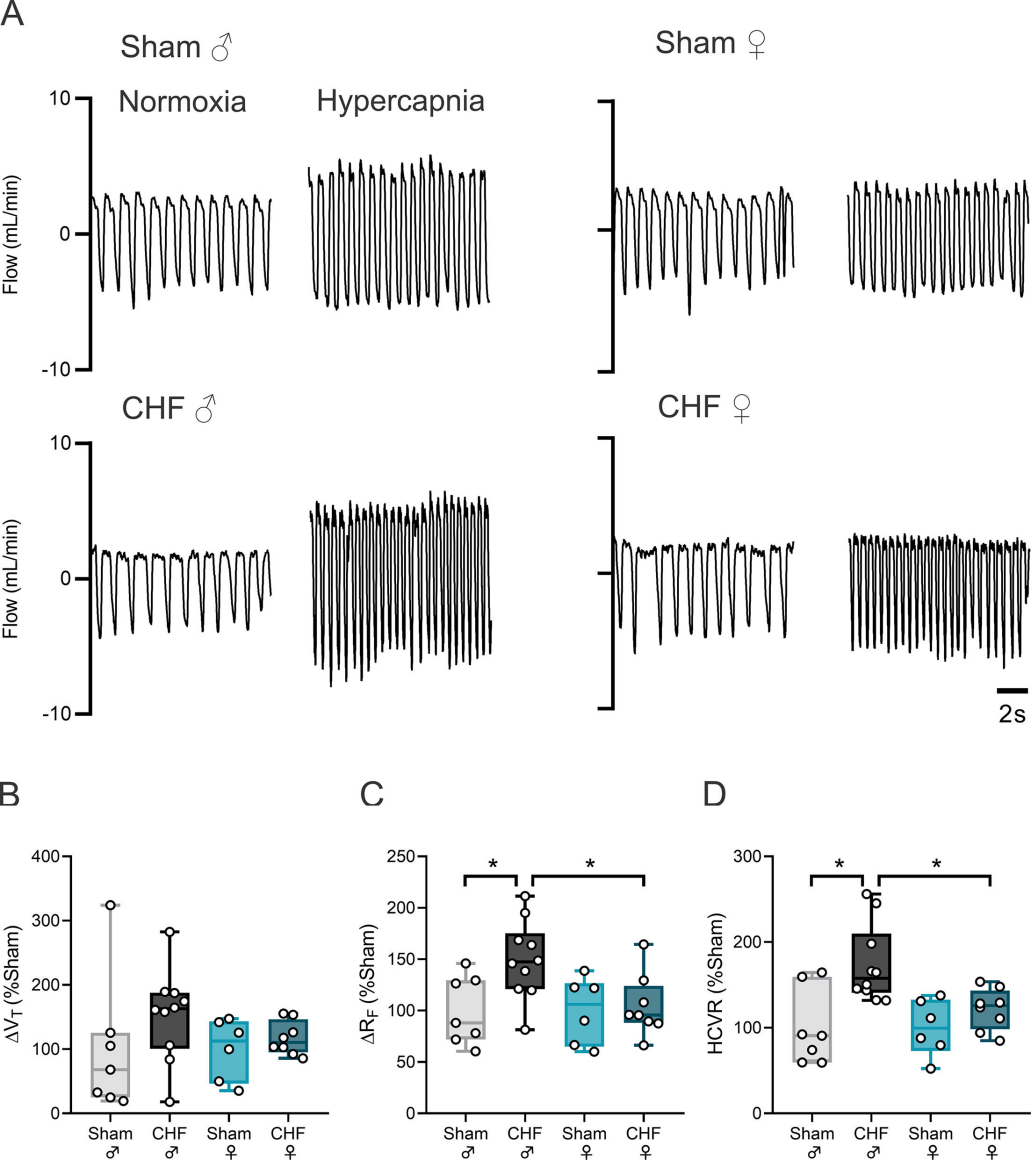
Central chemoreflex drive remains unchanged in female CHF animals. (**a**) Representative ventilatory traces after hypercapnia challenge (F_i_CO_2_ 7%) from one animal per group. (**b-d**) Summary data showing the magnitude of response in terms of tidal volume (V_T_), respiratory frequency (R_F_), and the hypercapnic ventilatory response (HCVR), respectively. Data were analyzed using One-way ANOVA and Holm–Sidak multiple comparisons test; *: *P*<0.05. *n* = 6–10 per group.

Male CHF rats display overt signs of irregular and disordered breathing (Figure 6a). Indeed, Poincaré plots show higher inter-breath interval variability and IS in CHF male rats compared with male Sham rats (Figure 6b and d–f). In addition, a higher oscillation in the magnitude of each ventilatory cycle was found in CHF male rats compared with Sham male rats (Figure 6g). Contrary to what we found in CHF male rats, females with CHF showed regular breathing patterns while breathing at eupneic conditions (Figure 6a–g). Indeed, IS was almost identical between Sham female rats and CHF female rats (Figure 6f). Lastly, CHF male rats showed a high incidence of disordered breathing (i.e. apneas, hypopneas) compared with Sham male rats (100.0 ± 14.29 vs. 225.0 ± 18.3, Sham male vs. CHF male), while CHF female showed no change in the incidence of apneas nor in hypopneas compared with Sham female rats (100.0 ± 13.5 vs. 125.4 ± 11.3, Sham female vs. CHF female). Since we previously reported a close link between disordered breathing and central chemoreflex drive following volume overload CHF, we assessed central chemoreflex drive in male and female rats with CHF (Figure 7). Compared with Sham groups, only males with CHF showed an increase in the HCVR. Indeed, a 1.7-fold increase in HCVR was found in male CHF rats compared with male Sham rats (100.0 ± 16.8 vs. 173.5 ± 14.3% Sham, Sham male vs. CHF male), while no differences were found between female CHF rats and female Sham rats (100.0 ± 16.8 vs. 121.6 ± 8.5, Sham female vs. CHF female) (Figure 7a, b and h). Interestingly, we found differences in HCVR between Sham males and Sham female rats, showing an increase in chemoreflex sensitivity in females compared with males (Table 5).

**Table 5:** Ventilatory chemoreflex function

	Sham ♂ (*n* = 7)	CHF ♂ (*n* = 10)	Sham ♀ (*n* = 6)	CHF ♀ (*n* = 8)
***Normoxia***				
V_t_ (ml/100 g)	0.2 ± 0.02	0.2 ± 0.01^ǂ^	0.4 ± 0.02	0.5 ± 0.03
R_f_ (bpm)	88.8 ± 2.0	92.3 ± 3.5^ǂ^	71.8 ± 4.5	72.2 ± 4.0
V_E_ (ml/min/100 g)	21.1 ± 1.9	23.1 ± 1.3	31.5 ± 3.9	34.2 ± 3.5
***Hypercapnia***				
V_t_ (ml/100 g)	0.3 ± 0.04	0.4 ± 0.02^ǂ^	0.7 ± 0.06	0.8 ± 0.06
R_f_ (bpm)	144.8 ± 7.5	176.1 ± 4.7*^ǂ^	134.8 ± 8.1	138.1 ± 6.2
V_E_ (ml/min/100 g)	45.2 ± 4.9	64.6 ± 2.9*^ǂ^	92.0 ± 7.9	107.9 ± 7.5

Values are means ± S.E.M. Two-way ANOVA followed by Holm–Sidak *post hoc* analysis. *P<0.05 vs. Sham male, †*P*<0.05 vs. female. Respiratory frequency, R_f_. Tidal volume, V_T._ Minute ventilation, V_E_. ^ǂ^ *P*<0.05 vs. CHF female. *P<0.05 vs. Sham male.

## Discussion

Epidemiological studies show that elderly women are more susceptible to CHF than men, potentially due to reduced cardio-protection associated with reproductive senescence. However, altered cardiovascular and respiratory reflexes, such as enhanced chemoreflex sensitivity and sympathoexcitation, could promote adverse cardiac remodeling, increased LV stiffness, and arrhythmogenesis independent of hormonal status [7–10]. Then, whether neural control of cardiorespiratory regulation may respond/adapt differentially in males versus females during the development of CHF is completely unknown. We aimed to determine the influence of sex (without the confounding of reproductive senescence/hormonal status) on pathophysiological hallmarks of cardiorespiratory function during volume overload CHF. Compared with males, we found that female rats: (i) developed only mild LV hypertrophy following volume overload, (ii) preserved cardiac function and cardiomyocyte Ca²^+^ homeostasis despite the increases in pre-load, (iii) maintained cardiac autonomic balance without any signs of sympathoexcitation following volume overload, (iv) displayed no pre-sympathetic motoneuron activation nor astrocyte reactivity within the RVLM region after volume overload, and (v) retained regular resting breathing and chemoreflex function. Thus, adult females did not develop the cardiorespiratory pathophysiological features of CHF despite equivalent volume overload.

Our findings align with previous studies showing that volume overload causes less severe effects in females, whereas males exhibit contractile dysfunction, increased mortality, and higher plasma catecholamines [32]. However, it expands current knowledge by showing that the RVLM region, a nodal point involved in cardiorespiratory alterations in heart failure, adjusts differently in males and females to the volume overload stimuli. Indeed, the fact that reactive astrocytes were found in males only (after volume overload) and that this correlated with heightened neuronal activity and cardiac sympathoexcitation suggests that the female RVLM may be partially protected by a mechanism encompassing medullary astrocytes.

There is substantial evidence supporting a role for reproductive hormones in sympathetic-mediated diseases. Of relevance to our study, estrogen signaling influences key brain regions involved in sympathetic regulation [33], and ovariectomized rodents exhibit elevated sympathetic tone that is reversed by estrogen replacement [34]. Although no studies have directly examined how estrogens, or other reproductive hormones, modulate RVLM function in CHF, it is plausible that hormonal influences in younger females help prevent hyperactivation of pre-sympathetic neurons during volume overload. An additional explanation for the blunted RVLM response in females may involve sex-dependent differences in heart-to-brain signaling during the early stages of volume-overload CHF. In our study, female hearts were more resilient to developing CHF despite being subjected to similar AV-shunt flows and thus comparable cardiac stress. Consequently, we cannot exclude the possibility that cardiac sensory signaling triggered by increased stretch differs between males and females and contributes to shaping RVLM output. Clarifying the mechanisms that protect the female RVLM from pathological activation during volume overload will require dedicated future investigations.

In male CHF models, C1 neurons in the RVLM are overactive and play a pivotal role in sympathoexcitation and ventilatory stability [14,35]. Indeed, ablation of these neurons improves cardiovascular function in males with CHF [11]. Here, we report for the first time that RVLM neuronal hyperactivity in CHF is also accompanied by significant changes in medullary astrocytes, as evidenced by significant shifts in astrocyte morphology towards more complex structures. Interestingly, phenotypic switches in astrocyte cell morphology have been closely linked to impaired neuron-to-glia communication and increased neuronal excitability in different brain regions [36]. Therefore, it is plausible to hypothesize that astrocytes from the RVLM became reactive in CHF, leading to impaired proper neuron/glia crosstalk, contributing to the development/maintenance of heightened neuronal activity, being the outcome sympathoexcitation. Whether the medullary astrocyte is activated before or during the onset of neuronal excitation in CHF remains to be determined.

In contrast to males, volume-overloaded CHF females showed no evidence of RVLM neuronal hyperactivation and no alterations in medullary astrocyte morphology. These findings suggest that the female RVLM may be protected from volume overload through mechanisms involving preserved neuron-to-glia communication. Supporting this notion, previous studies demonstrate that impaired neuro-glia signaling can modulate neuronal excitability in the female brain [36–40], and that estrogens play a pivotal role in maintaining proper neuron-to-glia communication [36,37,39,41]. However, estrogen is unlikely to be the only hormonal factor influencing neuro-glia interactions and autonomic regulation during CHF. Testosterone has also gained attention for its emerging relevance in CHF, as low testosterone levels have been associated with impaired ventilatory efficiency, heightened sympathetic activation, and poorer clinical outcomes [42,43]. These observations raise the possibility that fluctuations or deficiencies in testosterone may interact with other reproductive hormones at the level of the RVLM, leading to disrupted neuro-glia communication, enhanced sympathetic outflow, and increased cardiovascular vulnerability with aging. Future studies employing hormonal profiling, gonadectomy models, and hormone-replacement paradigms will therefore be critical to delineate the mechanistic contributions of reproductive hormones to RVLM function and to determine whether restoring or augmenting these pathways may offer therapeutic benefit in CHF.

In summary, CHF-related cardiorespiratory alterations involve complex neurohumoral pathways. Adult gonadally intact females are protected compared with intact males against volume-overload CHF, maintaining normal neural control or cardiorespiratory function. A potential cell-specific protection elicited by reproductive hormones on brainstem structures associated with autonomic/respiratory regulation in the setting of CHF deserves further investigation.

**Clinical Perspective**

- Given the underrepresentation of women in cardiovascular research and the limited exploration of sex-specific mechanisms in autonomic regulation, this study investigated sex differences in cardiorespiratory and cardiac function in chronic heart failure (CHF), independent of reproductive senescence.
- Gonadally intact adult females are protected against volume-overload CHF compared with intact males, exhibiting preserved cardiorespiratory function and reduced astrocytic remodeling with diminished chronic activation of rostral ventrolateral medulla catecholaminergic neurons, resulting in a less severe heart pathology.
- These results underscore the protective role of gonadally intact female sex in heart failure and point to sex-specific mechanisms as a basis for developing targeted approaches to prevent and treat cardiorespiratory dysfunction in women.

## Supplementary material

online supplementary figure 1.

## Data Availability

All data generated in the present study are included in this published article and in its online supplementary material. Data analysis is available from the corresponding author upon reasonable request.

## Open Access

This article has been published open access under our Subscribe to Open programme, made possible through the support of our subscribing institutions, learn more here: https://portlandpress.com/pages/open_access_options_and_prices#conditional

## Abbreviations

BP: blood pressure
C1: catecholaminergic
CHF: chronic heart failure
EDP: end-diastolic pressure
EDPVR: end-diastolic pressure–volume relationship
EF: ejection fraction
ESP: end-systolic pressure
ESPVR: end-systolic pressure–volume relationship
HCVR: hypercapnic ventilatory response
HR: heart rate
LV: left ventricular
LVEDD: LV end-diastolic diameter
LVESD: LV end-systolic diameter
RVLM: rostral ventrolateral medulla
SV: stroke volume
