# Supplementary material for: Neuro-glial mechanisms underlying sex-based resilience to cardiorespiratory dysfunction in heart failure
Source: Clin Sci (Lond). 2026 Jan 14;140(1):81–96. doi: 10.1042/CS20258455 (PMC12862954; doi:10.1042/CS20258455)
Supplement: online supplementary figure 1. [file cs-140-1-CS20258455-s001.docx]

**Supplementary Information**

**Neuro-Glial Mechanisms Underlying Sex-Based Resilience to Cardiorespiratory Dysfunction in Heart Failure**

Katherin V. Pereyra^1^*, Karla G. Schwarz^1^*, Esteban Díaz-Jara^1^, Sinay C. Vicencio^1^, Liena Bravo^1^, Fernando C. Ortiz^2^, Camilo Toledo^4^, Rodrigo Del Rio^1,3,4*^

^1^Laboratory of Cardiorespiratory Control, Department of Physiology, Pontificia Universidad Católica de Chile, Santiago, Chile. ^2^Mechanisms of Myelin Formation and Repair Laboratory, Departamento de Biología, Facultad de Química y Biología, Universidad de Santiago de Chile, Santiago, Chile. ^3^Center for Aging Research and Healthy Longevity, Faculty of Sciences, Universidad Mayor, Santiago, Chile. ^4^Department of Cell Biology and Physiology, School of Medicine, University of Kansas Medical Center, Kansas City, Kansas, United States.

**Short title:** Sex differences in heart failure

***** Katherin V. Pereyra and Karla G. Schwarz contributed equally to this work.

***Corresponding Author:** Rodrigo Del Rio, Ph.D.

Department of Cell Biology and Physiology

School of Medicine

University of Kansas Medical Center

3901 Rainbow Blvr HLSIC-2091

Kansas City, KS 66160, USA

E-mail: rdelrio@kumc.edu


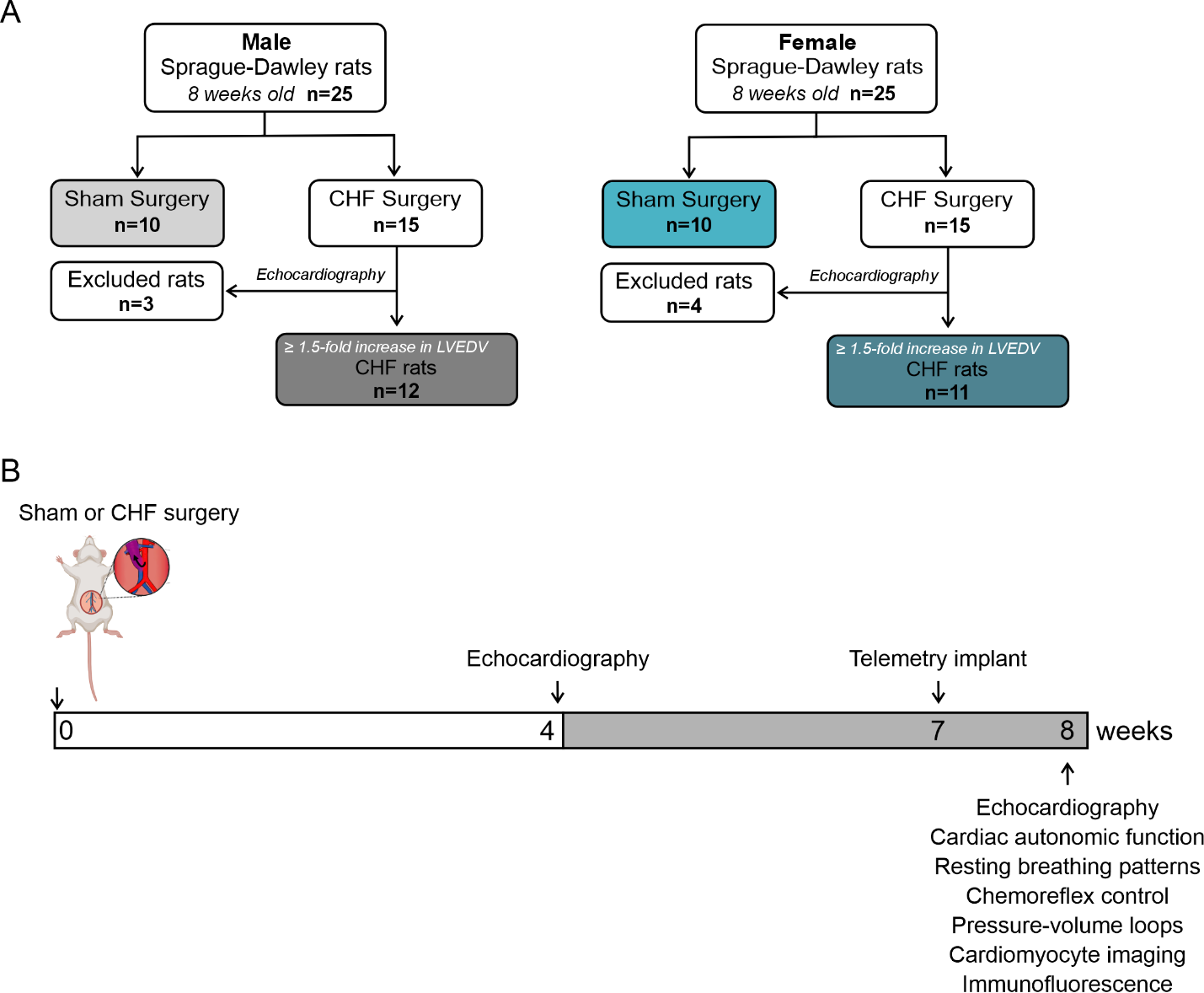


**Figure S1. Flow chart illustrating allocation of rats and experimental timeline.** (a) The study began with 50 adult Sprague–Dawley rats (8 weeks old): 25 male rats and 25 female rats. 10 rats of each sex underwent Sham surgery, whereas 15 rats of each sex underwent heart failure (CHF) surgery. 3 male and 4 female rats, which did not reach CHF criteria, were excluded. (b) Sham and CHF surgeries were performed at time 0. After 4 weeks, echocardiography was performed to confirm CHF condition. Physiological experiments were performed at 8 weeks post- Sham or CHF surgery.


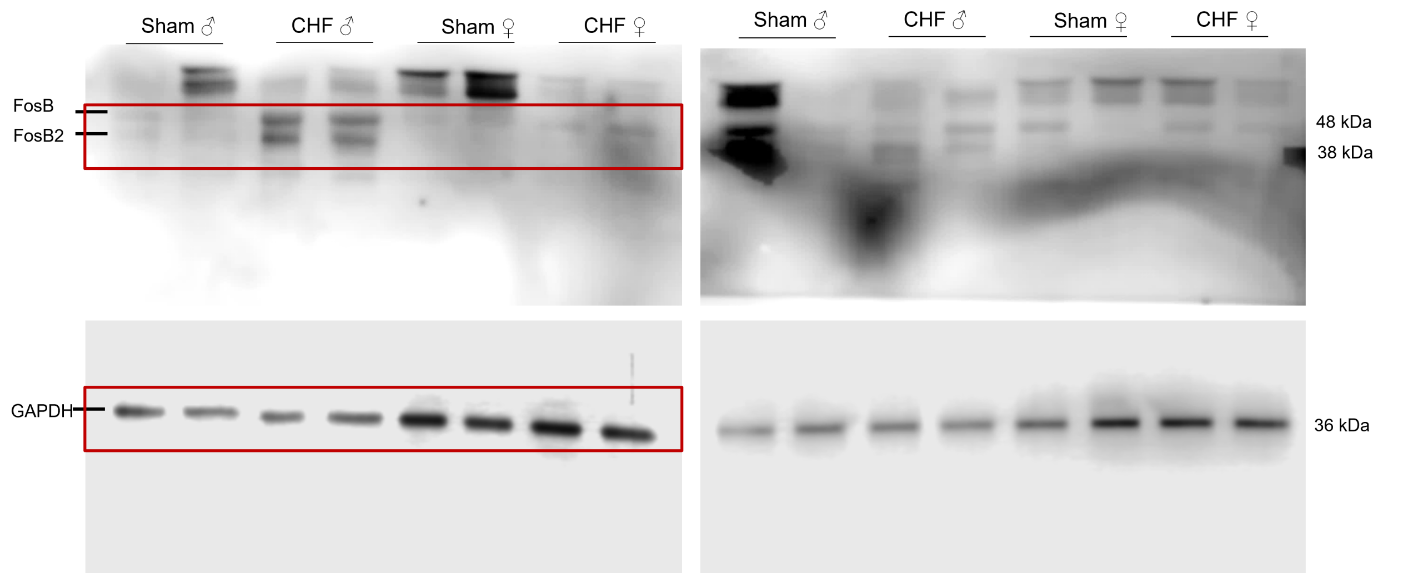


**Figure S2. FosB protein expression in Rostral Ventrolateral Medulla (RVLM).** 30 µg of protein extract RVLM of four rats of each experimental group were separated by SDS-PAGE. After electrotrasfer to a PVDF membrane (90mA, 16h, 4°C), the blotted bands were immunodetected with specific rabbit monoclonal anti-FosB (1:1000 on blocking buffer, Cell signaling) and mouse polyclonal anti-GAPDH (1:500 on blocking buffer, Santa Cruz Biotechnology) and subsequently visualized with peroxidase labeled anti-mouse IgG or anti-rabbit IgG antibodies. Red rectangle indicates the representative cropped blot shown in Figure 5 of the main manuscript.


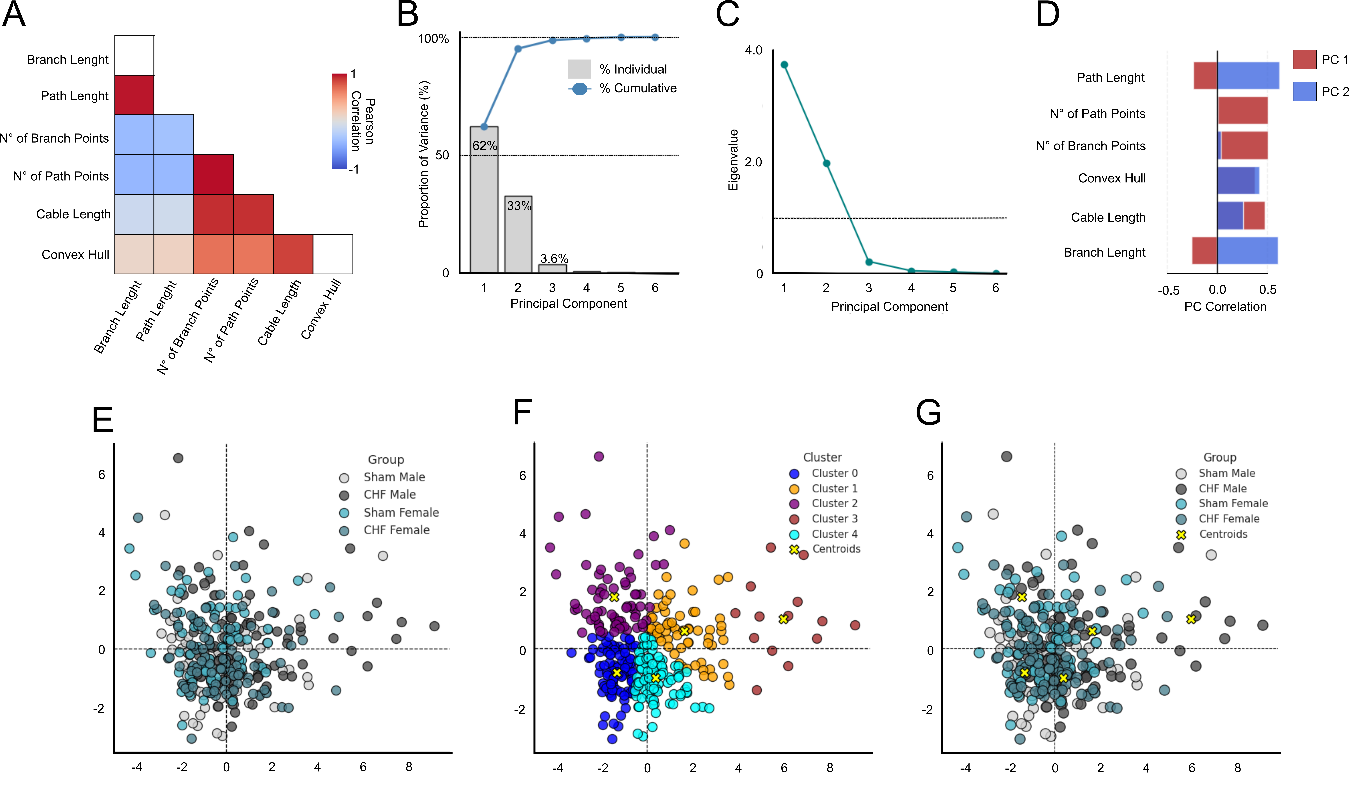
 **Figure S3. Multivariate analysis of astrocyte morphometric features reveals latent structural patterns and cluster identities.** (a) Correlation matrix of six extracted morphometric variables used for PCA: branch length, path length, number of branch points, number of path points, cable length, and convex hull boundary size. (b) Variance explained by each principal component (% individual and cumulative). PC1 and PC2 together account for 95% of the total variance. (c) Scree plot showing eigenvalues for each principal component; the inflection point supports the retention of two components. (d) Variable loading for PC1 and PC2. (e) PCA projection of all astrocytes colored by experimental group. (f) PCA projection colored by K-means cluster identity (five clusters). (g) PCA projection colored by experimental group, overlaid with cluster centroids used for complexity classification. n = 80–100 cells per group from 3-4 animals.
